# Comparative efficacy of erector spinae plane and quadratus lumborum blocks in managing postoperative pain for total abdominal hysterectomy: A randomized controlled trial

**DOI:** 10.1097/MD.0000000000040313

**Published:** 2024-10-25

**Authors:** Onur Baran, Ayhan Şahin, Cavidan Arar

**Affiliations:** a Department of Anesthesiology and Reanimation, Medical Faculty of Tekirdağ Namik Kemal University, Tekirdağ, Türkiye.

**Keywords:** anesthesia, erector spinae plane block, opioid consumption, postoperative pain, quadratus lumborum block, total abdominal hysterectomy

## Abstract

**Background:**

Effective postoperative pain management after total abdominal hysterectomy is crucial for patient recovery and satisfaction. This study compared the efficacy of the erector spinae plane block (ESPB) and quadratus lumborum block (QLB) in reducing postoperative opioid consumption and pain in patients with total abdominal hysterectomy.

**Methods:**

In this prospective, randomized controlled trial, 90 patients undergoing total abdominal hysterectomy were divided into 3 groups: ESPB, QLB, and control. The primary outcome was postoperative opioid consumption. Secondary outcomes included pain scores assessed by the visual analog scale at predetermined times and the incidence of postoperative nausea and vomiting. Statistical significance was determined using analysis of variance, the Mann–Whitney *U* test, and the Kruskal–Wallis test.

**Results:**

The ESPB and QLB groups showed a significant reduction in postoperative opioid consumption compared with the control group (*P* < .001 for both comparisons). Pain scores were significantly lower in the ESPB and QLB groups than in the control group at 2, 6, and 24 hours postoperatively (*P* < .001 at each time point). The incidence of postoperative nausea and vomiting was lower in the ESPB and QLB groups than that in the control group; however, this difference was not statistically significant (*P* = .029). No significant differences were observed in opioid consumption or pain scores between the 2 groups.

**Conclusion:**

Both the erector spinae plane and quadratus lumborum blocks effectively reduced postoperative opioid consumption and pain in patients with total abdominal hysterectomy. These techniques offer a promising approach for postoperative pain management, potentially reducing the need for opioids.

## 1. Introduction

Regarding frequency of application, cesarean section is the most commonly performed obstetric surgery, followed by hysterectomy.^[1,2]^ Total abdominal hysterectomy (TAH) is a serious major surgical procedure that negatively affects postoperative recovery and is associated with severe postoperative pain.^[3,4]^ Severe pain after TAH should be treated using a multimodal pain control strategy. Limiting opioid consumption, which has many side effects such as nausea, vomiting, and respiratory depression, and relieving pain by using the least amount of opioids is one of the most important goals of anesthesiologists.^[5]^

Although the quadratus lumborum block (QLB), an abdominal wall block applied under ultrasound guidance, was first defined by Blanco et al^[6]^ as a variation of the transversus abdominis plane block, various application approaches to this block have been defined over time.^[7,8]^ It is performed by applying a local anesthetic to the anterior, posterior, or lateral quadratus lumborum muscle, and the nomenclature is based on the targeted anatomical location.^[9]^ The anterior QLB is aimed at delivering high-volume local anesthetic to the interfascial plane between the quadratus lumborum muscle and the psoas muscle, and this approach is practical in providing postoperative pain control in many studies.^[9–12]^

Since its description by Forero et al,^[13]^ the erector spinae plane block (ESPB) has proven effective in providing postoperative analgesia and reducing opioid consumption after numerous surgical procedures.^[14–17]^ The main goal of this block is to block the ventral and dorsal branches of the relevant spinal nerves by applying a high volume of local anesthetic between the tip of the transverse process of the relevant vertebra and the erector spinae muscle under ultrasound guidance.^[18]^

In this study, we hypothesized that the ESPB and QLB could reduce opioid consumption by providing multimodal analgesia after TAH. In this context, the primary outcome was the 24-hour cumulative opioid consumption in the ESPB, QLB, and control groups, and the secondary outcomes were the postoperative visual analog scale (VAS) score, postoperative first rescue analgesic time, and the presence of nausea and vomiting.

## 2. Methods

This prospective, double-blind, randomized controlled study was conducted in accordance with the principles of the Declaration of Helsinki. The study protocol, approved by the Tekirdağ Namik Kemal University Ethics Committee, is registered with ClinicalTrials.gov on 09/01/2023 (NCT05675657). Written informed consent was obtained from all patients before their inclusion in the study.

Patients who were scheduled to undergo elective TAH under general anesthesia by the Department of Gynecology and Obstetrics at Tekirdağ Namik Kemal University Hospital, Tekirdağ, Türkiye between January 15, 2023, and July 15, 2023, were included in the study.

Patients aged 18 to 75 years with American Society of Anesthesiologists physical scores of I and II were included in the study. Patients who met the following exclusion criteria were excluded from the study: patients who did not agree to participate in the study, those with body mass index (BMI) > 35 kg/m^2^, patients younger than 18 years of age, patients older than 75 years of age, patients with uncontrolled systemic disease, those with whom cooperation could not be ensured, such as mental retardation, patients with low cardiac capacity, patients with a history of allergy to planned and possible drugs, coagulopathy, local infection, or addiction to opioids.

The patients were randomized into 3 groups using the sealed envelope method through a computer-based program (https://www.randomizer.org), with equal numbers of patients in each group. Researchers who applied the blocks to the patients and those who administered anesthesia and followed the patients during the postoperative period differed. Therefore, researchers other than those who applied the blocks to patients did not know which group the patients belonged to. The blinding was achieved in this manner.

All patients underwent surgery under general anesthesia. Before general anesthesia, the patients were evaluated in the preanesthesia room according to the group to which they were randomized. According to the sealed envelope system, the patients were taken directly to the operating room, and preparations for general anesthesia were initiated if the patients were in the control group. If the patients were in the ESPB or QLB group, they were placed in the area reserved for peripheral nerve blocks.

Patients underwent a 3-channel electrocardiogram, noninvasive blood pressure, and peripheral oxygen saturation monitoring in a particular area reserved for peripheral nerve block. A total of 0.02 mg kg^−1^ intravenous (iv) midazolam (Dormicum 5 mg/5 mL, Deva, İstanbul, Türkiye) was administered along with saline infusion sufficient to ensure patency of the intravenous vascular access. An ultrasound machine (Esaote, MyLab Six, Genova, Italy) with linear (13–3 MHz) and curved (8–1 MHz) probes was installed and used for the block groups.

All patients underwent surgery under general anesthesia. From the moment the patient entered the operating room, 3-channel electrocardiogram, noninvasive blood pressure, and peripheral oxygen saturation monitoring were performed. Following the induction with 2.5 mg kg^−1^ propofol (Propofol-pf %1 200 mg/20 mL, Polifarma, Ankara, Türkiye) 1 mcg kg^−1^ fentanyl (Talinat 0.5 mg/10 mL, Vem, İstanbul, Türkiye), and 0.5 mg kg^−1^ rocuronium (Esmeron 50 mg/5 mL, MSD, İstanbul, Türkiye), the patients were successfully intubated, and anesthesia was maintained with sevoflurane (Sevorane, Abbvie, İstanbul, Türkiye) and 50% oxygen–air mixture. All patients received a remifentanil (Ultiva 2 mg, Eczacibaşi, İstanbul, Türkiye) infusion titrated to 0.1 to 1 mcg kg min^−1^. Thirty minutes before the end of the surgery, 1 g of paracetamol (Parol 10 mg/mL, Atabay, İstanbul, Türkiye) and ondansetron (Kemoset 4 mg/2 mL, Deva, İstanbul, Türkiye) were administered. After successful recovery from anesthesia and extubation, the patients were transferred to a postanesthesia care unit (PACU).

A tramadol-based (Contramal 100 mg/2 mL, Abdi İbrahim, İstanbul, Türkiye) patient controlled analgesia device was connected to the patients in the PACU for postoperative analgesia. The patient controlled analgesia device contained 3 mg mL^−1^ tramadol (Contramal 100 mg/2 mL, Abdi İbrahim, İstanbul, Türkiye), was set to 3.5 mL bolus, had a 20-minute lockout, and had a 4-hour limit of 35 mL. During the postoperative period, 1 g of paracetamol (Parol 10 mg/mL, Atabay, İstanbul, Türkiye) was administered every 6 hours. In cases where the VAS score was 4 or above, diclofenac 75 mg (Dikloron 75 mg/3 mL, Deva, İstanbul, Türkiye) was ordered, followed by 100 mg tramadol (Contramal 100 mg/2 mL, Abdi İbrahim, İstanbul, Türkiye) as rescue analgesia when a VAS score of 4 or above was still observed 30 minutes later. Diclofenac (Dikloron 75 mg/3 mL, Deva, İstanbul, Türkiye) was administered at a maximum dose of 150 mg/day, and tramadol (Contramal 100 mg/2 mL, Abdi İbrahim, İstanbul, Türkiye) at a maximum of 300 mg/day. During this process, the VAS score, number of postoperative nausea and vomiting (PONV) episodes, and first rescue analgesic time were recorded in the PACU and ward at 2, 6, 12, and 24 hours by an assistant independent of the study.

### 2.1. ESPB technique

**Figure 1.**
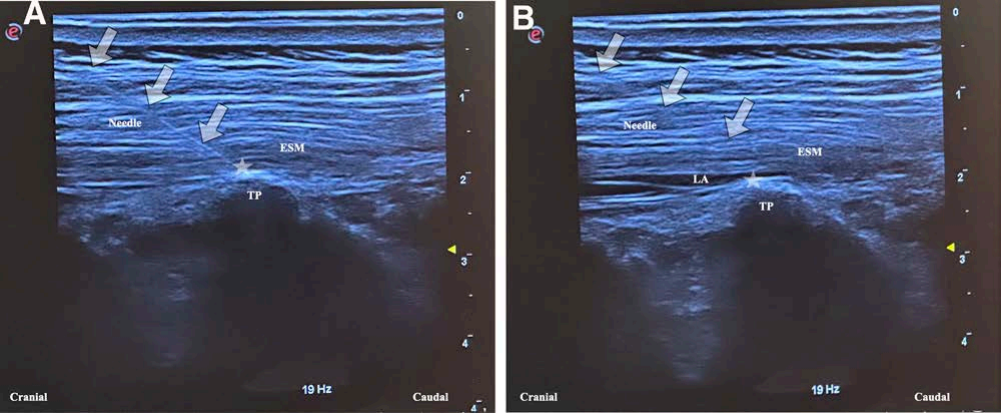
Erector spinae plane block. (A) A high-frequency linear ultrasound probe was placed craniocaudally 2 to 3 cm lateral to the midline at the T9 vertebra level. Using the in-plane technique, the needle was placed at the tip of the transverse process anterior to the erector spinae plane muscle with a craniocaudal approach. (B) The deposition of local anesthetic into the plane between the tip of the transverse process and the erector spinae muscle. ESM = erector spinae muscle, LA = local anesthetic, TP = transverse process. The star indicates the tip of the needle. White arrows indicate the needle shaft.

For ESPB, the patient was placed in the prone position. After skin sterilization, a high-frequency linear ultrasound probe was placed perpendicular to the midline at the T9 vertebra level. After the spinous process level was identified on the ultrasound screen, the probe was rotated 90° clockwise or counterclockwise, such that the identifying light remained on the cranial side and shifted 2 to 3 cm laterally from the midline. The paraspinal anatomical structures were identified, and the transverse process and erector spinae muscle were observed posteriorly. A 20-gauge 100 mm sonovisible peripheral nerve block needle (Ultraplex 360, B-Braun, Melsungen, Germany) was advanced craniocaudally towards the transverse process under ultrasound guidance, keeping the needle tip visible at all times. When the needle tip touched the transverse process, it was slightly withdrawn, and 1 to 2 mL of saline was administered for testing (Fig. 1A). If it was observed that the interfascial plane between the erector spinae muscle and the transverse process was separated, 30 mL bupivacaine (Marcaine 0.5%, AstraZeneca, İstanbul, Türkiye) at 0.25% concentration was given in divided doses and intermittently with negative aspiration, if no blood was observed (Fig. 1B). The same procedure was performed on the contralateral side at the same vertebral level under the same conditions.

### 2.2. QLB technique

**Figure 2.**
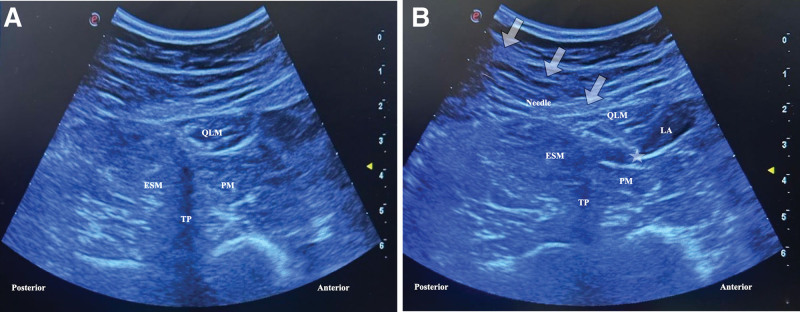
Quadratus lumborum block. (A) The transverse processes of the L4 vertebra, psoas, quadratus lumborum, and erector spinae muscles were detected with a low-frequency curvilinear probe placed between the iliac crest and the 12th rib at the midaxillary line. (B) Local anesthetic spread was observed in the plane between the quadratus lumborum and psoas muscles. ESM = erector spinae muscle, LA = local anesthetic, TP = transverse process. QLM = quadratus lumborum muscle. The star indicates the tip of the needle. White arrows indicate the needle shaft.

For the QLB, the patient was first placed in a lateral decubitus position with the side chosen for the block on top. By ensuring all aseptic conditions, the transverse processes of the L4 vertebra, psoas muscle, quadratus lumborum, and erector spinae muscles were detected with a low-frequency curvilinear probe placed between the iliac crest and the 12th rib at the midaxillary line (Fig. 2A). Using a 20-gauge 100 mm sonovisible peripheral nerve block needle (Ultraplex 360, B-Braun, Melsungen, Germany) under ultrasound guidance, keeping the needle tip constantly visible, the quadratus lumborum muscle was passed transmuscularly in the posteroanterior plane, and 1 to 2 mL of saline was administered to the interfascial plane between the psoas muscle and the quadratus lumborum muscle, following negative blood aspiration. After the separation in the interfascial plane was observed, 30 mL 0.25% bupivacaine (Marcaine 0.5%, AstraZeneca, İstanbul, Türkiye) was applied with intermittent aspiration to prevent intravenous injection. Local anesthetic spread was observed between the quadratus lumborum and psoas muscle (Fig. 2B). The same procedure was performed on the contralateral side, under identical conditions.

### 2.3. Control group interventions

In the control group, patients were transferred from preanesthesia to the operating room without block interventions.

### 2.4. Outcome measurements

The primary outcome was the 24-hour cumulative opioid consumption in the ESPB, QLB, and control groups. The secondary outcomes were the postoperative visual analog scale score, postoperative first rescue analgesic time, and the presence of nausea and vomiting, recorded in the PACU and ward at 2, 6, 12, and 24 hours by an assistant independent of the study.

### 2.5. Sample size calculation

The power analysis for our primary hypothesis, which investigated “the effect of the ESPB and QLB on postoperative opioid consumption in patients undergoing TAH,” was conducted with a focus on the variable of postoperative opioid consumption to determine whether there was a significant effect between different treatment groups.

Owing to the lack of directly comparable studies in the literature and the inability to conduct a pilot study, the power analysis was based on the assumptions of a medium effect size (Cohen d of 0.5), 5% risk of Type I error (alpha), and 80% power (1-beta error, Type II error).

The analysis concluded that approximately 14 participants per group (ESPB, QLB, and control) were required, making 42 participants sufficient. The sample size was determined to ensure adequate power to test the primary hypotheses. However, our study included 90 participants (30 in each group).

### 2.6. Statistical analysis

Descriptive statistics were used to summarize the data. For continuous (numerical) variables, depending on the distribution, either mean ± standard deviation or median with minimum and maximum values were presented in a tabular format. Categorical variables are summarized as counts and percentages. The normality of the numerical variables was assessed using the Shapiro–Wilk, Kolmogorov–Smirnov, and Anderson–Darling tests.

The Fisher–Freeman–Halton test was used to compare categorical variable differences across groups. Compared to 2 independent groups, the Mann–Whitney *U* test was used for numerical variables that did not follow a normal distribution.

In comparisons involving more than 2 independent groups, the one-way analysis of variance (ANOVA) test was used for numerical variables that exhibited a normal distribution, while the Kruskal–Wallis *H* test was employed for those that did not exhibit a normal distribution. For multiple comparisons in parametric tests, either the Games–Howell or Tukey tests were used, whereas for nonparametric tests, the Dwass–Steel–Critchlow–Fligner test was applied.

For within-group comparisons of pulse, mean arterial pressure, resting VAS score, and movement VAS score, the repeated measures ANOVA was used when numerical variables showed a normal distribution, and the Friedman test, a nonparametric version of repeated measures ANOVA, was employed otherwise.

The Tukey test was used for parametric test methods, whereas the Durbin–Conover test was applied for nonparametric methods to identify differences between measurements. This study incorporated the Bonferroni correction to control for the possibility of false positives (Type I error) due to multiple comparisons. The Bonferroni correction was used to reduce the risk of false determination of significance in each of the multiple statistical tests.

Our study measured 2 variables across 3 groups (ESPB, QLB, and control). These measurements were taken at 6 distinct time points to assess pulse and mean arterial pressure: preanesthetic, immediately post-induction, at the 1st-hour post-induction, at the 2nd-hour post-induction, at the end of the case, and in the PACU. Additionally, measurements were conducted at 4 different time points in the same 3 groups for the resting and moving VAS scores.

We performed all possible pairwise comparisons among the 3 groups at each time point, resulting in 3 pairwise comparisons at each time point (ESPB–QLB, ESPB–control, and QLB–control). Consequently, with measurements repeated across 6 time points, the pairwise comparisons totaled 18 for pulse and mean arterial pressure. Similarly, for the resting and moving VAS scores, 12 pairwise comparisons were made, considering 3 pairwise comparisons across the 4 time points.

After applying Bonferroni correction, the overall significance level of 0.05 was divided by 18 comparisons. Consequently, the new threshold for the significance of each comparison was set to *P* < .0028. A *P*-value below this threshold indicated statistical significance for the respective comparison.

As mentioned above, the Bonferroni correction and the newly calculated significance threshold of *P* < .0028 were explicitly applied to the results presented in Tables 2 and 3. These adjustments and thresholds are specific to the multiple time points, and group comparisons in this table and are not applicable to the analysis results in other study sections.

**Table 2:** Comparative heart rate and mean arterial pressure analysis among the ESPB, QLB, and control groups.

	Groups			*P*
	ESPB (n = 30)	QLB (n = 30)	Control (n = 30)	
Pulse (bpm)∥				
Preanesthetic	80.5 [56.0–109.0]	88.5 [66.0–121.0]	79.0 [50.0–110.0]	.007†
Immediate post-induction	78.0 [55.0–109.0]	87.5 [70.0–116.0]	84.0 [56.0–124.0]	.003†
1st hour post-induction	75.0 [50.0–95.0]	81.5 [61.0–94.0]	72.5 [57.0–104.0]	.061†
2nd hour post-induction	76.5 [54.0–96.0]	80.5 [63.0–95.0]	75.0 [62.0–99.0]	.281†
End of case	81.0 [60.0–96.0]	76.5 [61.0–92.0]	75.0 [62.0–116.0]	.970†
PACU	76.5 [59.0–99.0]	74.0 [63.0–95.0]	69.5 [50.0–99.0]	.132†
*P*§	.035	**<.001**	.120	
Mean arterial pressure (mm Hg)¶				
Preanesthetic	98.8 ± 13.7	93.4 ± 11.4	104.3 ± 13.2	.005*
Immediate post-induction	87.5 ± 11.2	90.8 ± 9.9	93.0 ± 14.6	.245*
1st hour post-induction	83.6 ± 9.3	83.5 ± 7.4	88.3 ± 9.8	.089*
2nd hour post-induction	84.6 ± 12.5	85.2 ± 9.1	90.6 ± 13.4	.266*
End of case	94.7 ± 11.9	91.2 ± 11.1	94.1 ± 14.6	.462*
PACU	94.3 ± 12.5	94.3 ± 11.8	97.6 ± 10.1	.403*
*P*‡	**<.001**	**<.001**	**<.001**	

Comparison of the heart rate and mean arterial pressure (MAP) among the erector spinae plane block (ESPB), quadratus lumborum block (QLB), and control groups in a clinical setting. *P*-values indicate the statistical significance of the differences observed, with values <.05. The table offers a comprehensive view of temporal changes in heart rate, mean arterial pressure among the groups throughout the perioperative period.

* Statistical tests included one-way analysis of variance (ANOVA). These tests evaluate differences across the groups at various time points: preanesthesia, immediately post-induction, hourly intraoperatively, at the end of the case, in the postanesthesia care unit (PACU).

† Statistical tests included the Kruskal–Wallis test. These tests evaluate differences across the groups at various time points: preanesthesia, immediately post-induction, hourly intraoperatively, at the end of the case, in the postanesthesia care unit (PACU).

‡ Statistical tests included the repeated measures ANOVA. These tests evaluate differences across the groups at various time points: preanesthesia, immediately post-induction, hourly intraoperatively, at the end of the case, in the postanesthesia care unit (PACU).

§ Statistical tests included the Friedman test. These tests evaluate differences across the groups at various time points: preanesthesia, immediately post-induction, hourly intraoperatively, at the end of the case, in the postanesthesia care unit (PACU).

∥ The heart rate is represented by median values.

¶ Mean arterial pressure is shown as mean ± standard deviation.

**Table 3:** Comparative analysis of resting and movement visual analog scale (VAS) scores among the ESPB, QLB, and control groups.

	Groups			*P*
	ESPB (n = 30)	QLB (n = 30)	Control (n = 30)	
Resting VAS score (0–10)‡				
Postoperative 2nd hour	1.0 [0.0–3.0]	2.0 [0.0–3.0]	2.0 [1.0–4.0]	**<.001** *
Postoperative 6th hour	1.5 [0.0–3.0]	2.0 [0.0–3.0]	2.0 [1.0–4.0]	.009*
Postoperative 12th hour	1.5 [0.0–3.0]	2.0 [0.0–3.0]	2.0 [1.0–3.0]	.089*
Postoperative 24th hour	1.0 [0.0–3.0]	1.0 [0.0–3.0]	2.0 [1.0–4.0]	**.002** *
*P*†	.149	.677	.729	
Movement VAS score (0–10)‡				
Postoperative 2nd hour	2.0 [0.0–3.0]	2.0 [0.0–3.0]	3.0 [1.0–4.0]	**<.001** *
Postoperative 6th hour	2.0 [1.0–3.0]	2.0 [1.0–3.0]	3.0 [2.0–4.0]	**<.001** *
Postoperative 12th hour	3.0 [1.0–3.0]	2.5 [1.0–3.0]	3.0 [1.0–4.0]	**.002** *
Postoperative 24th hour	2.0 [0.0–3.0]	2.0 [0.0–3.0]	3.0 [1.0–4.0]	**<.001** *
*P*†	.013	.228	.996	

Comparison of the resting and movement visual analog scale (VAS) scores among the erector spinae plane block (ESPB), quadratus lumborum block (QLB), and control groups in a clinical setting. *P*-values indicate the statistical significance of the differences observed, with values <.05. The table offers a comprehensive view of temporal changes in heart rate, mean arterial pressure, and pain perception among the groups throughout the perioperative period, as assessed using VAS scores.

* Statistical tests included one-way analysis of variance (ANOVA). These tests evaluate differences across the groups at various time points: 2nd, 6th, 12th, and 24th hours in the ward postoperatively.

† Statistical tests included the Friedman test. These tests evaluate differences across the groups at various time points: 2nd, 6th, 12th, and 24th hours in the ward postoperatively.

‡ VAS scores are represented by median values.

Statistical analyses were performed using Jamovi (version 2.3.28) and JASP (version 0.17.3) software, and a significance level of .05 (*P*-value) was considered for all statistical analyses.

## 3. Results

**Figure 3.**
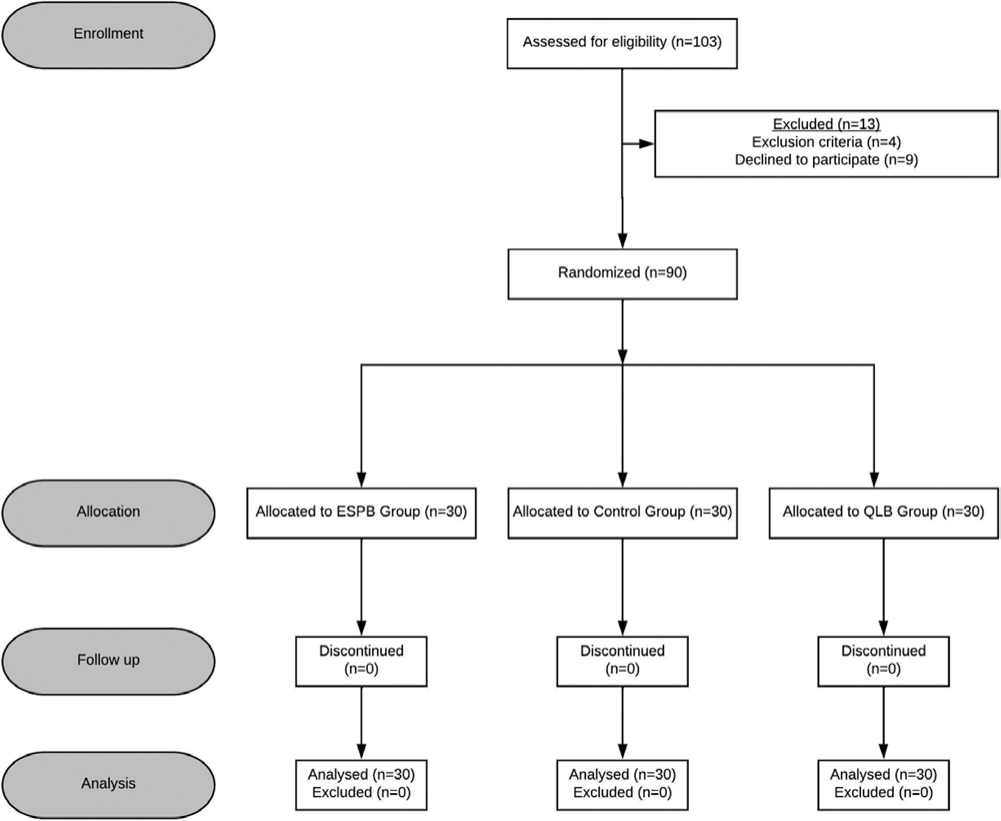
Consolidated Standards of Reporting Trials (CONSORT) flow diagram. ESPB = erector spinae plane block, QLB = quadratus lumborum block.

In our study, 103 patients were screened, and 13 were excluded (Fig. 3). Ninety participants were analyzed; the mean age was 47.7 ± 6.1 years. The mean BMI of the participants was 25.6 ± 3.1. The median block time for the procedure was 480 seconds. The median operative time was 122 minutes. Postoperative pain assessed using the PACU VAS, had a median score of 2. The total amount of postoperative opioids administered had a median of 52.5 mg. The median time to first rescue analgesic requirement was 16 hours. Regarding PONV, most patients (76.7%, n = 69) did not experience adverse events.

The study revealed no significant differences between the groups in terms of BMI, block time, or operative time (*P* > .05). However, a significant age disparity was observed, with patients undergoing the ESPB being significantly older than those receiving the QLB (*P* = .003). Regarding postoperative pain management, both the PACU VAS scores and total postoperative opioid analgesic doses were comparable between the ESPB and QLB groups (*P* > .05). Conversely, these measurements were significantly higher in the control group than in the ESPB and QLB groups (*P* < .05). The time to the first rescue analgesic requirement was significantly shorter in the control group than in the ESPB and QLB groups (*P* < .001 and *P* = .001, respectively). Simultaneously, it was similar between the ESPB and QLB groups (*P* = .978). Lastly, the incidence of PONV was comparable between the ESPB and QLB groups but significantly higher in the control group (*P* = .029) (Table 1).

**Table 1:** Comparison of demographic and clinical characteristics among ESPB, QLB, and control groups in a surgical patient population.

	Overall (n = 90)	Groups			*P*
		ESPB (n = 30)	QLB (n = 30)	Control (n = 30)	
Age (yr)∥	47.7 ± 6.1	49.9 ± 6.9	45.2 ± 3.6	48.1 ± 6.5	**.003** *
BMI (kg/m^2^)∥	25.6 ± 3.1	25.9 ± 3.4	25.8 ± 2.6	25.1 ± 3.2	.583*
Block time (s)¶	480.0 [147.0–900.0]	480.0 [147.0–600.0]	488.5 [300.0–900.0]	–	.212†
Operation time (min)¶	122.0 [60.0–300.0]	134.5 [70.0–220.0]	132.5 [85.0–200.0]	120.0 [60.0–300.0]	.530‡
PACU VAS score¶	2.0 [0.0–4.0]	1.0 [0.0–3.0]	1.0 [0.0–3.0]	2.0 [1.0–4.0]	**.001** ‡
Postoperative total opioid (mg)¶	52.5 [10.5–105.0]	42.0 [21.0–63.0]	52.5 [10.5–63.0]	63.0 [31.5–105.0]	**<.001** ‡
First rescue analgesic need hour (h)¶	16.0 [1.0–23.0]	19.0 [13.0–23.0]	19.0 [13.0–23.0]	4.5 [1.0–11.0]	**<.001** ‡
Number of PONV¶	0.0 [0.0–1.0]	0.0 [0.0–1.0]	0.0 [0.0–1.0]	0.0 [0.0–1.0]	**.030** ‡
No**	69 (76.7)	26 (86.7) ††	25 (83.3) ††	18 (60.0) ‡‡	**.029** §
Yes**	21 (23.3)	4 (13.3) ††	5 (16.7) ††	12 (40.0) ‡‡	

Comparison of the demographic and clinical characteristics among erector spinae plane block (ESPB), quadratus lumborum block (QLB), and control groups in a surgical patient population. “††” and “‡‡” indicate significant group differences identified in multiple comparison tests, providing insights into variations in factors such as age, body mass index (BMI), block time, operation duration, postanesthesia care unit (PACU) visual analog scale (VAS) score, total postoperative opioid consumption, time first to rescue analgesic requirement, and number of postoperative nausea and vomiting (PONV) episodes. *P*-values indicate the statistical significance of the differences observed, with values <.05.

* Statistical analyses included one-way analysis of variance (ANOVA) to evaluate differences across the ESPB, QLB, and control groups

† Statistical analyses included Mann–Whitney *U* test to evaluate differences across the ESPB, QLB, and control groups

‡ Statistical analyses included Kruskal–Wallis test to evaluate differences across the ESPB, QLB, and control groups

§ Statistical analyses included Fisher–Freeman–Halton test to evaluate differences across the ESPB, QLB, and control groups.

∥ Mean ± standard deviation.

¶ Median values with ranges [minimum–maximum].

** The number and percentage of patients (n [%]).

Our study found no significant differences between the groups in terms of pulse rate and mean arterial pressure at any measurement point (both *P* > .0028). In the measurements of resting VAS scores, significant differences emerged between the groups at the postoperative 2nd and 24th hours (*P* < .001 and *P* = .002, respectively). Specifically, the VAS scores were significantly higher in the control group than in the ESPB group at the postoperative 2nd hour. No significant differences were observed in the other pairwise comparisons. At the postoperative 24th hour, there were no significant differences in the pairwise comparisons between the groups. When analyzing the movement VAS scores, significant differences were observed at all measurement points between the groups (*P* < .0028 for each). Movement VAS scores at the postoperative 2nd, 6th, and 24th hours were notably higher in the control group than in both the ESPB and QLB groups (*P* < .001), while scores were similar between the ESPB and QLB groups (*P* > .05). At the postoperative 6-h mark, no significant differences were found in the pairwise comparisons of the movement VAS scores (each *P* > .028).

**Figure 4.**
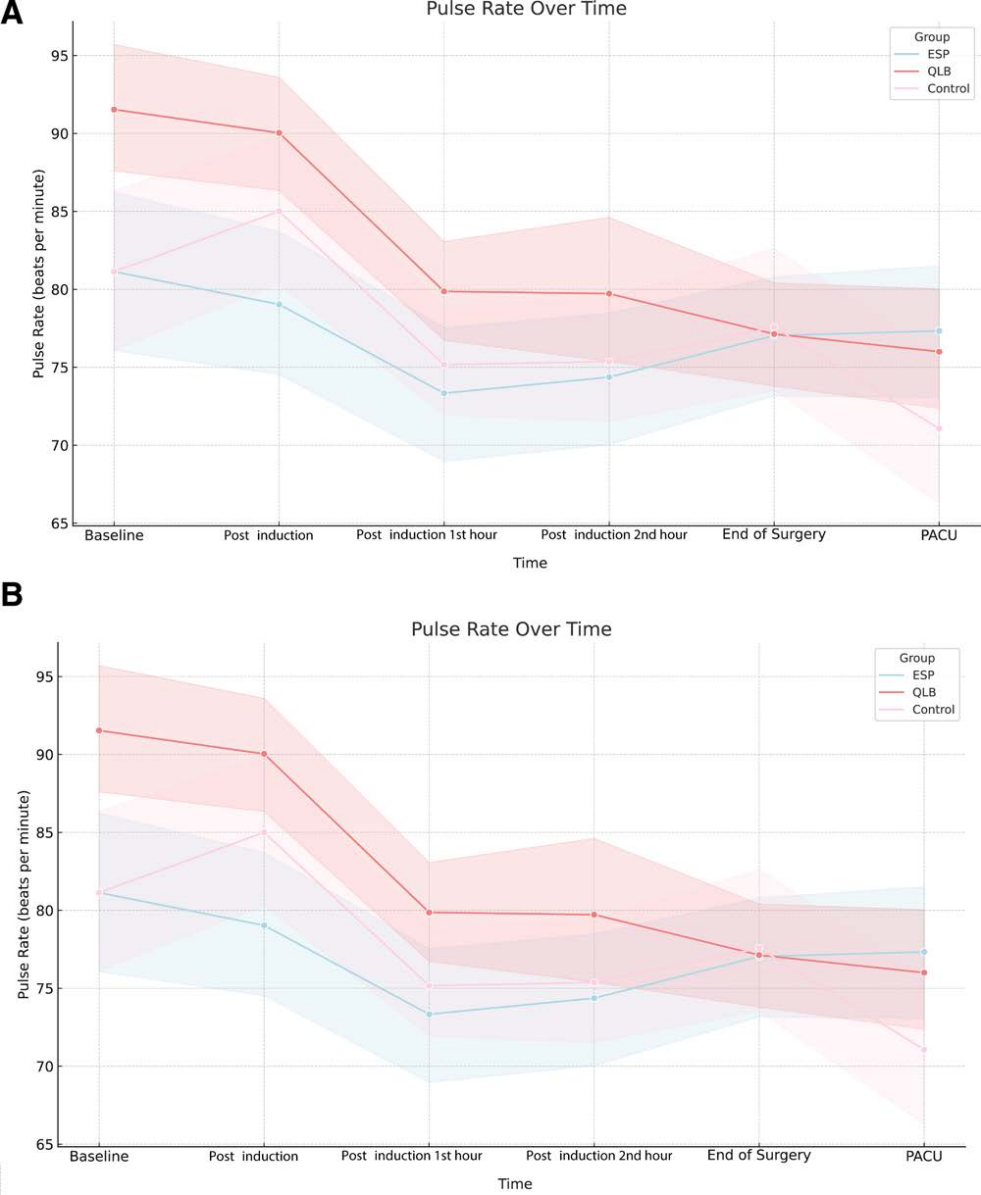
(A) Illustrates the variation in pulse rate among diverse groups over time, and (B) depicts the trends in mean arterial pressure within erector spinae plane block (ESPB), quadratus lumborum block (QLB), and control groups over the same period.

**Figure 5.**
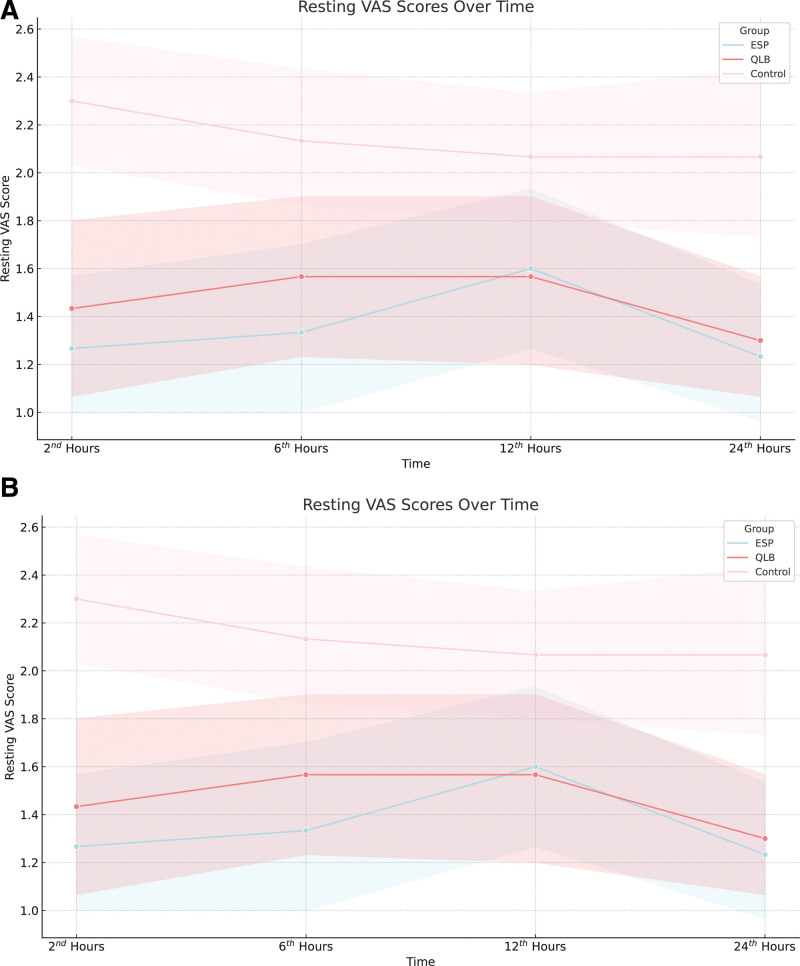
(A) Illustrates the variation in resting visual analog scale (VAS) scores among the different groups over time, and (B) depicts the trends in movement VAS scores within the erector spinae plane block (ESPB), quadratus lumborum block (QLB), and control groups over the same period.

In the intragroup comparisons within the ESPB group, no significant changes were observed in the pulse rate and resting and movement VAS scores (each *P* > .0028). However, a significant change was observed in the mean arterial pressure over time (*P* < .001). Specifically, the mean arterial pressures at the 1st and 2nd hour post-induction were significantly lower than the baseline values (each *P* < .001). No significant differences were observed in the other pairwise comparisons within the ESPB group (each *P* > .0028). No significant changes in the pulse rate and resting or movement VAS scores were observed in the control group (each *P* > .0028). However, a significant difference was found in mean arterial pressure (*P* < .001), with immediate post-induction mean arterial pressures being significantly lower than the preanesthetic values (*P* < .001) (Figs. 4 and 5) (Tables 2 and 3).

## 4. Discussion

Our study’s primary focus was to evaluate the analgesic efficacy of the ESPB and QLB in patients undergoing TAH, with particular emphasis on postoperative opioid consumption. The results indicated that both ESPB and QLB were effective in reducing postoperative pain and opioid requirements compared with the control group, with no significant difference between the ESPB and QLB groups.

These findings are consistent with those of recent studies that have explored the effectiveness of ESPB and QLB in various surgical contexts. For instance, Jiang et al^[19]^ demonstrated that both the ESPB and the transmuscular QLB improved multimodal analgesia quality in total laparoscopic hysterectomy, suggesting their potential to reduce opioid consumption. Similarly, our study found comparable efficacy between ESPB and QLB, reinforcing that both techniques are viable options for postoperative pain management in patients with TAH.

Moreover, the study by Zanfini et al^[20]^ on postoperative analgesia after cesarean section using ESPB and QLB found no significant difference in total morphine consumption between the 2 groups, aligning with our findings of comparable opioid consumption between ESPB and QLB groups. These findings further support the idea that both blocks effectively manage postoperative pain during abdominal surgery.

Interestingly, a study on laparoscopic liver resection^[21]^ also reported similar postoperative analgesia between the ESP and QL blocks, which aligns with our findings. This consistency across different types of surgeries suggests a broader applicability of these blocks in various surgical procedures.

Furthermore, a study comparing the ESPB and QLB in open nephrectomy^[22]^ reported similar outcomes in terms of morphine consumption and pain scores, corroborating our findings regarding the efficacy of both blocks.

A study on pediatric postoperative pain management^[23]^ suggested that the QLB might provide more effective analgesia than the ESPB in specific contexts, indicating the need for further research to explore the differential effectiveness of these blocks in various patient populations and surgical procedures.

In terms of the adjuvants used in ESPB, a study^[24]^ highlighted the efficacy of dexmedetomidine over dexamethasone in enhancing the analgesic profile of ESPB. This finding suggests potential avenues for optimizing ESPB techniques, which could be relevant to future studies on TAH.

In a study conducted by Abdelaziz et al,^[25]^ 64 female patients undergoing abdominal hysterectomies were compared with ESPB and QLB, and ESPB was reported to be a more effective, simple, and safe approach to multimodal analgesia.

Finally, a comparison between the QLB and transversus abdominis plane block^[2,26]^ in patients with TAH showed that the QLB was more effective, reinforcing our study’s findings regarding the efficacy of the QLB in managing postoperative pain in TAH.

This study had some certain limitations. Even though the study was conducted prospectively and randomly, the patients were awake even under sedation during the block procedures. Although they did not know which block was performed, the patients may not be considered fully blinded. This situation was tried to be eliminated by ensuring that the people who followed the patients in the postoperative period did not know whether any blocks were applied to the patients. Although the blocks performed under ultrasound were performed by experienced hands, this study may be limited by its inability to objectively evaluate the quality of the blocks.

## 5. Conclusions

Our study contributes significantly to the evidence supporting the ESPB and QLB as effective postoperative pain management strategies for patients with TAH. These findings align with existing literature, suggesting that both ESPB and QLB are viable options for reducing opioid consumption and managing postoperative pain during abdominal surgery. The current place of fascial plane blocks in multimodal analgesia and their success in reducing opioid use may change our approach in clinical practice. Future studies should explore the differential effects of these blocks on various patient demographics, surgical procedures, and the use of different adjuvants to optimize their analgesic efficacy.

## Acknowledgments

The authors would like to express special appreciation and thanks to Gökhan Karakoç for statistical consultation.

## Author contributions

**Conceptualization:** Onur Baran, Ayhan Şahin.

**Data curation:** Onur Baran, Ayhan Şahin.

**Formal analysis:** Onur Baran, Ayhan Şahin.

**Investigation:** Onur Baran.

**Methodology:** Onur Baran, Ayhan Şahin, Cavidan Arar.

**Software:** Onur Baran.

**Supervision:** Cavidan Arar.

**Writing – original draft:** Onur Baran, Ayhan Şahin, Cavidan Arar.

**Writing – review & editing:** Onur Baran, Ayhan Şahin, Cavidan Arar.
